# Boosting Formate Production from CO_2_ at High Current Densities Over a Wide Electrochemical Potential Window on a SnS Catalyst

**DOI:** 10.1002/advs.202004521

**Published:** 2021-05-29

**Authors:** Jinshuo Zou, Chong‐Yong Lee, Gordon G. Wallace

**Affiliations:** ^1^ ARC Centre of Excellence for Electromaterials Science Intelligent Polymer Research Institute AIIM Innovation Campus University of Wollongong Wollongong NSW 2500 Australia

**Keywords:** alkaline, CO_2_ reduction, flow‐cell, formate, tin sulfide

## Abstract

The flow‐cell design offers prospect for transition to commercial‐relevant high current density CO_2_ electrolysis. However, it remains to understand the fundamental interplay between the catalyst, and the electrolyte in such configuration toward CO_2_ reduction performance. Herein, the dramatic influence of electrolyte alkalinity in widening potential window for CO_2_ electroreduction in a flow‐cell system based on SnS nanosheets is reported. The optimized SnS catalyst operated in 1 m KOH achieves a maximum formate Faradaic efficiency of 88 ± 2% at −1.3 V vs reversible hydrogen electrode (RHE) with the current density of ≈120 mA cm^−2^. Alkaline electrolyte is found suppressing the hydrogen evolution across all potentials which is particularly dominant at the less negative potentials, as well as CO evolution at more negative potentials. This in turn widens the potential window for formate conversion (>70% across −0.5 to −1.5 V vs RHE). A comparative study to SnO*x* counterpart indicates sulfur also acts to suppress hydrogen evolution, although electrolyte alkalinity resulting in a greater suppression. The boosting of the electrochemical potential window, along with high current densities in SnS derived catalytic system offers a highly attractive and promising route toward industrial‐relevant electrocatalytic production of formate from CO_2_.

## Graphical abstract

The use of KOH electrolyte in a flow‐cell configuration is found to boost the electrochemical potential window for CO_2_ electroreduction to formate on a SnS catalyst. The significant suppression of H_2_ and CO evolutions over a wide potential range leads to high formate conversion efficiencies at high current densities.

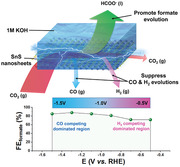

## 1 Introduction

The burning of fossil‐fuels as energy sources to support increasingly demanding industries and household activities over the past decades has resulted in the alarming level of anthropogenic CO_2_ gas in the atmosphere.^[^ ^1^ ^]^ Electrochemical reduction of CO_2_ offers a viable clean energy technology to mitigate CO_2_ by converting this greenhouse gas to valuable chemical feedstocks.^[^ ^2^ ^]^ Among the liquid products, formic acid or formate has been found wide‐ranging applications in chemical industries for production of household products,^[^ ^3^ ^]^ as well as offering promise as a safe liquid‐phase chemical for hydrogen storage and conversion.^[^ ^4^ ^]^ In comparison to other formate producing CO_2_ electrocatalysts, such as bismuth,^[^ ^5^ ^]^ indium,^[^ ^6^ ^]^ lead,^[^ ^7^ ^]^ and palladium,^[^ ^8^ ^]^ the low cost, eco‐friendly and nontoxic characteristics of tin make it an outstanding and promising catalyst candidate for CO_2_ reduction to formate.^[^ ^9^ ^]^

To overcome the intrinsically poor electrocatalytic CO_2_ reduction performance of a bulk Sn with a large overpotential and low current density, various material engineering strategies have been applied. This includes nanostructuring of Sn to structures such as metallic Sn quantum sheets,^[^ ^10^ ^]^ coralline structured SnO*x*,^[^ ^11^ ^]^ SnO_2_ porous nanowires,^[^ ^12^ ^]^ ultrasmall SnO,^[^ ^13^ ^]^ and SnS_2_ nanosheets,^[^ ^14^ ^]^ that promotes surface area and active sites toward selective formate production. In addition to alloying or doping with metals, such as copper,^[^ ^15^ ^]^ silver,^[^ ^16^ ^]^ palladium,^[^ ^17^ ^]^ nickel,^[^ ^18^ ^]^ positive synergetic impact of doping of a nonmetallic element, such as sulfur^[^ ^19^ ^]^ or nitrogen^[^ ^20^ ^]^ is another attractive approach toward enhancing formate production. From experimental and density functional theory results, sulfur was found played a role in stabilizing the *OCHO intermediate, weakening the interaction between CO and electrode, and therefore favoring the formation of formate.^[^ ^14^, ^19^, ^21^ ^]^ However, there are still limitations with respect to generally low operating current density, as well as the narrow electrochemical potential window available to ensure high formate Faradaic efficiency.

Recent studies suggest that the use of a flow‐cell capable of supplying gaseous CO_2_ from the backside of a gas diffusion electrode (GDE) to react with the electrocatalyst, overcomes the limitation of poor CO_2_ solubility (0.034 m) in an aqueous solution when employing a laboratory H‐cell configuration.^[^ ^22^ ^]^ Flow‐cells have recently been employed for electrochemical CO_2_ reduction reaction (CO_2_RR) to overcome the low current densities by reducing the mass transfer issue that exists in the H‐cell system, as the tri‐phasic solid/liquid/gas interfaces maximize the interaction between the catalysts and the reactants.^[^ ^23^ ^]^ In this configuration, the selectivity of a catalyst can be tuned by employing an alkaline KOH or NaOH electrolyte. For example, Dinh et al. reported improved ethylene selectivity to 70% via KOH‐mediated CO_2_ electrocatalysis on a copper catalyst.^[^ ^24^ ^]^ Irtem et al.^[^ ^22a^ ^]^ applied the flow‐cell using Sn based catalyst which obtain formate conversion Faradaic efficiency as high as 71% for 6 h, though the current density was low (8.58 mA cm^−2^ at −1.1 V vs RHE). Liang et al.^[^ ^25^ ^]^ improved the total current density (147 mA cm^−2^ at −0.95 V vs RHE) and tuned the selectivity of hydrocarbon and oxygenate (C_2_H_5_OH) by changing the electrolyte (KOH vs KHCO_3_) on ultrasmall SnO_2_ nanoparticles. This system shows formate Faradaic conversion efficiency of ≈75% with a narrow potential window of around −0.73 V vs RHE.

It is desirable if the non‐noble metallic catalyst, such as Sn is able to be developed to achieve the high formate conversion efficiency, as well as operability over a wide range of potential windows for electrochemical CO_2_ reduction. Boosting the cathodic potential window offers an advantage of the flexibility in coupling to anodic reactions. This in turn will allow full‐cell CO_2_ electrolyser operable at a wider operation potential range. In this study, we synthesized and optimized tin‐based catalyst, SnS nanosheets, to be an efficient electrocatalyst for formate production. This followed by rationally investigating conditions that allows operation at a wide potential window, as well as promoting the SnS electrocatalyst long‐term stability. We also examined mechanistic aspects of the role of alkalinity in widening the electrochemical potential window.

## 2 Results and Discussion

### 2.1 Preparation and Characterization of SnS/Gas Diffusion Layer (SnS/GDL)

**Figure 1:**
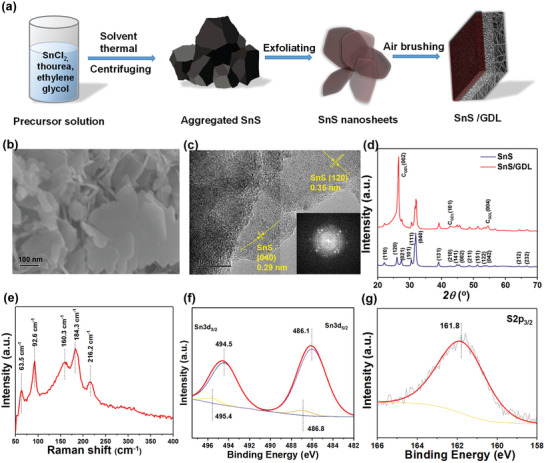
a) The schematic diagram on the steps to fabricate SnS/GDL; SEM b) and TEM c) image of SnS, with inset in c) is the corresponding fast Fourier transform (FFT) image taken from the TEM image. d) X‐ray diffraction (XRD) patterns of SnS and SnS/GDL, e) Raman spectrum of SnS/GDL, and f,g) XPS spectra of Sn and S elements on SnS/GDL.

**Figure** **1**a schematically illustrates the preparation of the SnS catalyst loaded on a gas diffusion layer (GDL). The SnS was first formed by a modified solvothermal method by treating the precursors SnCl_2_ and thiourea in the solvent of ethylene glycol at the temperatures between 140 and 200 °C for 12 h. After washing several times by centrifuging in ethanol, the bulk SnS was exfoliated by ultrasonication for 1 h in isopropanol to obtain a homogenous SnS nanosheet suspension.^[^ ^26^ ^]^ Then the ink containing SnS nanosheets, isopropanol and nafion solution was air‐brushed on a commercially available carbon paper‐based GDL (Sigracet 39 BC) with the catalyst loading of 0–2 mg cm^−2^ and dried at 50 °C overnight. More experimental details are described in the Experimental Section. The X‐ray diffraction (XRD) patterns and the scanning electron microscope (SEM) images of the catalysts synthesized at different temperatures are shown in Figure 1b,1d; and Figures S1 and S2 (Supporting Information). As presented in Figures S3 and S4 (Supporting Information), it was found the SnS catalyst obtained at solvothermal temperature of 180 °C, with the SnS loading of 1 mg cm^−2^ to be the optimal conditions. Therefore, unless otherwise stated, all the characterization and performance results are based upon the SnS/GDL samples prepared under the above conditions.

The SEM images in Figure 1b; and Figure S2 (Supporting Information) reveal the synthesized SnS nanosheets with sheet sizes of 0.1–1 µm and a thickness of 10 ± 2 nm. The lattice structure of SnS was further characterized by transmission electron microscopy (TEM) (Figure 1c). The SnS lattice fringes with d‐spacing of 0.35 and 0.29 nm are from SnS (120) and SnS (040), respectively. The XRD patterns of SnS and SnS/GDL confirming the crystalline phase of SnS with predominant diffraction peaks in Figure 1d at 22.0°, 27.4°, 30.5°, 31.9°, 39.0° are originated from orthorhombic SnS crystal facets (JCPDS 39‐0354), which is in agreement with the *d*‐spacing in Figure 1c. The Raman spectroscopy spectrum of the SnS/GDL recorded at the wavelength of 50–400 cm^−1^ is presented in Figure 1e. Raman modes at 63.5, 92.6, 160.3, 184.3, 216.2 cm^−1^ are consistent with the signature optical phonons modes of SnS.^[^ ^27^ ^]^ The peaks at 184.3 and 216.2 cm^−1^ represent the longitudinal optical A*_g_* (LO) modes, whereas 92.6 cm^−1^ belongs to the transverse optical A*_g_* (TO) mode. The mode at 63.5 cm^−1^ is assigned to the combination of A*_g_* and B_2_ *_g_* of SnS, while the mode at 160.3 cm^−1^ can be attributed to B_2_ *_g_* of SnS phase. Importantly, there is no SnS_2_ or Sn_2_S_3_ associated peaks that are expected to be at around 312 and 308 cm^−1^, suggesting that the obtained tin sulfide is a pure SnS phase. Composition and valence state analyses were conducted by X‐ray photoelectron spectroscopy (XPS) spectrum of Sn 3d (Figure 1f). There are two main peaks at 486.1 and 494.5 eV corresponding to Sn3d_5/2_ and Sn3d_3/2_.^[^ ^28^ ^]^ The XPS fittings of small peaks at 486.8 and 495.4 eV represent the Sn^4+^, potentially attributed to natural oxidation of Sn^2+^ in air. The Sp_3/2_ peak at 161.8 eV in Figure 1g corresponds to S^2−^ species attached to Sn^2+^.^[^ ^28a^ ^]^

### 2.2 CO_2_ Electroreduction Performance

**Figure 2:**
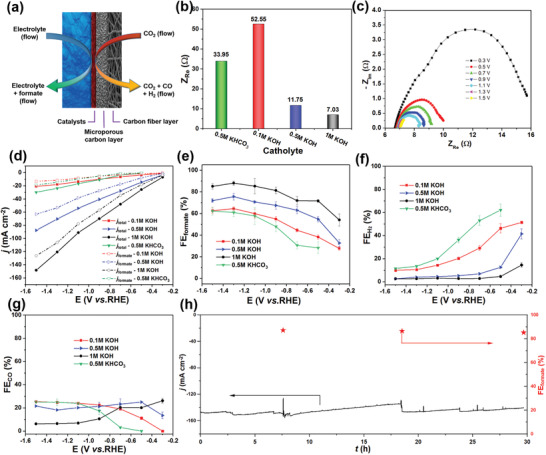
a) Schematic of the cathodic part of flow‐cell configuration. b) Ohmic resistance of SnS/GDL samples in different electrolytes obtained from the Nyquist impedance plots in Figure S8b (Supporting Information) with the electrode potential of −1.5 V vs RHE. c) Nyquist impedance plots of SnS/GDL sample at different potentials from −0.3 to −1.5 V vs RHE in the catholyte of 1 m KOH. All the Nyquist impedance plots were obtained in frequency range from 1 to 100 mHz with 30 mV amplitude. d) Steady‐state current density of SnS/GDL samples in different electrolytes, solid symbols represent the total current density (*j*_total_), while empty symbols represent the partial current density of formate (*j*_formate_). e–g) Faradic efficiencies toward formate, H_2_ and CO obtained in 0.5 m KHCO_3_, 0.1 m KOH, 0.5 m KOH, 1 m KOH. The error bars represent the standard deviations of three independent measurement of the sample. (h) Stability test of SnS/GDL at −1.5 V vs RHE. The experiments were conducted in the catholyte of 1.5 L of 1 m KOH.

The CO_2_RR performance tests were conducted in a three‐electrode flow electrochemical cell (**Figure** **2**a), a photograph of the experimental setup is shown in Figure S5 (Supporting Information). The synthesized SnS was homogenously loaded on the GDL consisting of a microporous carbon layer on a macroporous carbon fiber layer to serve as the working electrode (Figure S6, Supporting Information). In this tri‐phasic solid/liquid/gas system, the CO_2_ gas flowed through the carbon layers to reach the SnS catalyst at a flow rate of 20 mL min^−1^. The catholyte and anolyte were circulated into the corresponding compartments at a flow rate of 17.5 mL min^−1^.

We investigated CO_2_ electroreduction using the SnS/GDL with different electrolytes: 0.5 m KHCO_3_ and KOH with concentrations from 0.1 to 1.0 m. As shown in the linear sweep voltammogram (LSV) curves in Figure S7 (Supporting Information), the current densities increased significantly with the use of more concentrated KOH electrolyte, which is largely attributed to the drop of ohmic resistance from 52.6 *Ω* (0.1 m KOH) to 7.0 *Ω* (1 m KOH) (Figure 2b; and Figure S8a, Supporting Information), meanwhile solution resistance of 0.5 m KHCO_3_ is 33.9 *Ω*. Noted that an identical trend of ohmic resistance was observed at −1.5 V vs RHE and under the open circuit voltage (see Figure S8b, Supporting Information). In addition to the ohmic resistance information, potential‐dependent Nyquist impedance plots offer valuable information on the charge‐transfer resistance that reflecting the electrocatalytic activity. The impedance plots at a low frequency region for SnS/GDL in Figure 2c reveals the drop in charge transfer resistance (smaller semicircles) with the increase of applied cathodic potentials from −0.3 to −0.5 V vs RHE, correlated well with the enhancement in electrocatalytic current. Likewise, greater driving force at more negative applied potentials (−0.5 to −1.5 V) results in lower charge‐transfer resistances.

Controlled potential electrolysis (CPE) experiments were performed at fixed potentials of between −0.3 and −1.5 V vs RHE. The gaseous products from CPE experiments, CO and H_2_, were quantified by gas chromatography (GC), whereas formate (a liquid product) was determined using nuclear magnetic resonance (NMR). The plots of total current density (*j*_total_) and the partial current density of formate (*j*_formate_) based on geometrical surface area are presented in Figure 2d and Figure S9 (Supporting Information), showing an almost linear increment with increased cathodic potentials. The *j*_total_ and *j*_formate_ were normalized by electrochemical surface area (ECSA) (Figures S10 and S11, Supporting Information) employing the ECSA value of 115.75 cm^2^. As is consistent with Figure S7 (Supporting Information), increased KOH concentration shows higher current densities. *j*_total_ in 1 m KOH is ≈7.1 and ≈1.7 times larger than that in 0.1 and 0.5 M KOH, whereas *j*_formate_ in 1 m KOH is ≈9.7 and ≈2.0 times of that in 0.1 and 0.5 m KOH at −1.5 V. In 0.5 m KHCO_3_, the *j*_total_ and *j*_formate_ are relatively low, almost at the same level of those in 0.1 m KOH.

CPE Data in Figure 2e and Figure S12 (Supporting Information) shows that 1 m KOH electrolyte corresponds to highest CO_2_ to formate conversion Faradaic efficiency (FE_formate_) across the applied potentials in comparison to other electrolytes. In this electrolyte, even at the potential of −0.3 V vs RHE, which is considered as a relatively positive potential for Sn‐based electrocatalysts, a FE_formate_ of 54.1 ± 6% can be reached. The maximum FE_formate_ of 88 ± 2% was obtained at −1.3 V vs RHE. Maximum FE_formate_ was also observed at this potential for other KOH electrolytes: 0.5 m KOH (75.7 ± 2%) and 0.1 m KOH (64.5 ± 2%). In addition to the maximum FE_formate_ as commonly reported as a figure of merits for CO_2_ reduction benchmark efficiencies, an interesting feature observed from this study is a wide potential range was achieved with efficiency over 70% from −0.5 to −1.5 V vs RHE. This performance to our best knowledge has not been reported before for any SnS‐based catalysts for CO_2_ electroreduction to formate.

To shed some light on factors contributing to the Faradaic conversion efficiencies, H_2_ and CO are plotted in Figure 2f,g, respectively. FE_H2_ was found to be dominant at the less negative potentials, −0.3 to −0.9 V, especially in 0.5 m KHCO_3_. The use of KOH, with increased concentrations from 0.1 to 1.0 m, drastically suppresses the H_2_ production, e.g., FE_H2_ dropping from 46.3 ± 4% (in 0.1 m KOH) to 4.6 ± 1% (in 1.0 m KOH) FE_H2_ at −0.5 V vs RHE. To further understand the suppression of hydrogen evolution of SnS during CO_2_ reduction, a control experiment was performed to compare the partial current density of H_2_, *j*_H2_, under the flow of CO_2_ and Ar gases in 1 m KOH electrolyte (Figure S13, Supporting Information). In Ar atmosphere, hydrogen gas (eg. *j*_H2_ = ≈90 mA cm^−2^ at −1.5 V vs RHE) with unity Faradaic efficiency was detected. In CO_2_ atmosphere, both CO_2_ reduction and hydrogen evolution occur with *j*_H2_ significantly suppressed across a wide potential range (e.g., *j*_H2_ < ≈4 mA cm^−2^ from −0.3 to −1.5 V vs RHE). This result highlights the significant of CO_2_ as a reactant competing with protons in the electrolyte. Although both the formation of formate and hydrogen need protons, the formate production is a preferable pathway under CO_2_ atmosphere. Across all the electrolytes, the CO by‐product contributes less than 30% Faradaic conversion efficiency. At more negative potentials from −0.9 to −1.5 V, it is found CO was suppressed to ≈7% in 1.0 m KOH. Hence, we proposed that the suppressions of both H_2_ and CO by‐products have contributed to an overall wider operational potential range. The Tafel slopes for SnS in both 1 m KOH and 0.5 m KHCO_3_ (see Figure S14, Supporting Information) are close to the theoretical value of 118 mV dev^−1^, indicating the one electron transfer forming CO_2_^−^ intermediates is the rate determining step (RDS).^[^ ^29^ ^]^ Comparatively, SnS in KOH (124 mV dev^−1^) has improved kinetics as it exhibits a lower Tafel slope than that in KHCO_3_ (155 mV dev^−1^).

It should be noted that we also performed CO_2_RR in 2 m KOH, however, SnS nanosheets were unstable as they gradually dissolved over time in this highly concentrated alkaline, evidently from the dropped in current from CPE study (Figure S15, Supporting Information). Noted also control experiment performed on the employed bare GDL, did not exhibit CO_2_ reduction products such as CO and formate, except H_2_, as a result of proton reduction reaction. The stability test of SnS/GDL was conducted in 1.5 L of 1 m KOH catholyte for 30 h (Figure 2h). Noted that this large volume of electrolyte reservoir was employed as it was found the electrolyte volume can affect the current density as the carbonate or bicarbonate formed during the CO_2_ gas purging can reduce the current density and a large volume of KOH electrolyte could help to mitigate this effect (see Discussion in Figure 4. After a 30 h test, the current density remained at 140.3 mA cm^−2^, only dropped 4%. The FE_formate_ decreased from 87% to 85%, indicating the excellent stability of CO_2_ reduction to formate. To evaluate catalyst stability during electrolysis, we performed structural and composition characterization of samples after 1 and 30 h CO_2_ electroreduction, respectively. The XRD, SEM, and XPS results are shown in Figure S16 (Supporting Information). After 1 h electrolysis, the nanosheets structure remained and the SnS was a dominant phase. However, after 30 h electrolysis, the surface morphology indicated aggregation of nanosheets with metallic Sn becoming the dominant phase. Such phase transition during electrolysis whereby some SnS is reduced to metallic Sn is consistent with the literature reports.^[^ ^19^, ^30^ ^]^ As the current density and formate selectivity remained relatively stable, suggesting that catalyst active sites are not significantly affected by the alteration in the surface morphology and phase transition within the performed experimental time‐scale.

**Figure 3:**
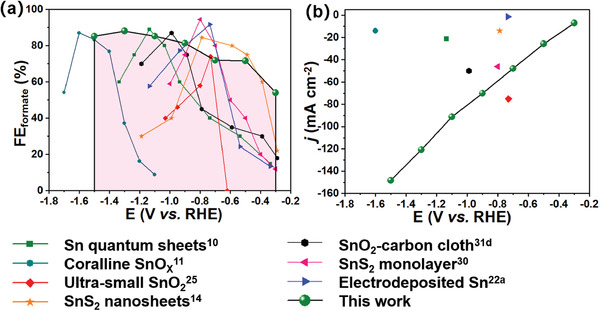
Summary of comparison between the reported Sn‐based catalyst and the current work for electrochemical CO_2_ reduction. a) FE_formate_ as a function of applied cathodic potentials, and b) The obtained current densities at the maximum FE_formate_ as a function of applied cathodic potentials. The potentials were converted to RHE scale based on the equations: E (vs RHE) = E (vs Ag/AgCl) + 0.0591 * pH + 0.21 and E (vs RHE) = E (vs SCE) + 0.0591 * pH + 0.24. It was assumed that the pH values of CO_2 –_ saturated 0.1 and 0.5 m NaHCO_3_/KHCO_3_ aqueous solution were 6.8 and 7.2, respectively.

A detailed performance comparison between this work and other published Sn‐based electrocatalysts for CO_2_ reduction to formate is presented in **Figure** **3** and Table S1 (Supporting Information).^[^ ^10^, ^11^, ^12^, ^13^, ^14^, ^22^, ^25^, ^30^, ^31^ ^]^ The potential window across −0.5 V to −1.5 V with FE_formate_ over 70% obtained from this work is clearly broader than literature studies that typically display “volcano shape” characteristic. Li et al.^[^ ^14^ ^]^ reported SnS_2_ nanosheets that having a potential window of −0.4 to −0.8 V, but having a low current density (<20 mA cm^−2^). In contrast, although ultrasmall SnO_2_ reported by Liang et al.^[^ ^25^ ^]^ has a high current density of 147 mA cm^−2^ at −0.95 V vs RHE, the FE_formate_ – E curve shows a narrow operating potential window. Other tailored catalysts such as Sn quantum sheets/Graphene,^[^ ^10^ ^]^ SnO_2_ nanosheets on carbon cloth,^[^ ^31d^ ^]^ SnS_2_ monolayer,^[^ ^30^ ^]^ and electrodeposited Sn^[^ ^22a^ ^]^ have either low current density or narrow operating potential window. It is important to note that all studies in literatures are based on the H‐cell configuration, except ultrasmall SnO_2_^[^ ^25^ ^]^ and electrodeposited Sn.^[^ ^22a^ ^]^ This work outperforms in achieving both high current density and high Faradaic efficiency for formate over a broad potential window. The three‐phase boundary, namely gaseous CO_2_, liquid KOH, and solid phase of SnS catalysts in this flow‐cell configuration promoting high current density, as well as allowing alkalinity impact to take place in suppressing competitive H_2_ and CO evolutions.

### 2.3 Understanding the Impact of Catholyte Volume Toward Current Stability

**Figure 4:**
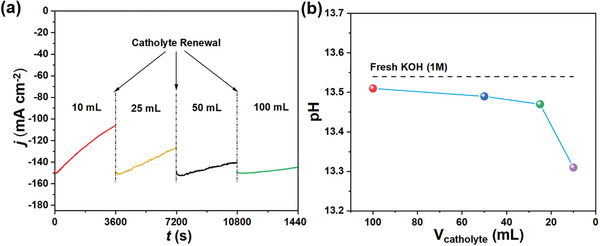
Effect of catholyte volume. a) *j*–*t* curves of SnS/GDL sample with different catholyte volumes. The experiments were conducted at the potential of −1.5 V vs RHE. b) The pH values of the catholyte after 1 h test in Figure 4a

To understand the impact of volume of KOH electrolyte reservoir on current stability, CO_2_RR performance was studied by replacing fresh batches of 1 m KOH catholyte with increasing volumes from 10 to 100 mL after consecutive 1 h study on the same SnS/GDL sample. In a small 10 mL KOH reservoir, the current density was found to decrease by 29% from 150 to 106 mA cm^−2^. In contrast, a high current stability was achieved with only 4% drop from an initial current when 100 mL KOH was employed. From **Figure** **4**a, it is important to note that the current density recovered to initial current of ≈150 mA cm^−2^ when a fresh catholyte was introduced, suggesting the current deactivation is a reversible process. Another important observation is despite the dropped in current densities, the impact on Faradaic conversion efficiencies of CO_2_ reduction products is negligible (Figure S17a, Supporting Information).

We further investigated the sources that cause the drop in current density. Since formate is the main product, it may be adsorbed on SnS and make the catalyst deactivated. To examine this possibility, we intentionally added 1 mL of 2.3 m formate in 50 mL catholyte every 10 min (Figure S17b, Supporting Information). Each addition of 1 mL formate solution provides equivalent amount of formate generated over 1 h electrocatalysis in 1 m KOH at −1.5 V vs RHE. However, there is a negligible change in the current density, indicating formate does not affect the current density. The second possibility might be the gaseous product, CO, adsorbed on the electrode surface, as reported in other catalyst such as Pd.^[^ ^32^ ^]^ We employed the method reported by Kanan and co‐workers to investigate potential CO effect.^[^ ^33^ ^]^ The 1 m KOH in cathodic chamber was emptied after 1 h electrocatalysis, and kept for 5 min to ensure any adsorbed CO on SnS electrocatalyst was readily purged and removed by the continuous flow of CO_2_. In the subsequent 1 h electrocatalysis using the same electrolyte, the current density was not recovered but continuously dropped (Figure S17c, Supporting Information). We ruled out CO as the cause of deactivation since the gas‐phase CO would have been purged and removed, and current density would have returned to the initial value if CO causes the deactivation.

We then investigated the final possible reason, the formation of carbonate or bicarbonate, which leads to pH change during the CO_2_ electrolysis. The continuously CO_2_ was purging at 20 mL min^−1^ through the backside of GDE to the SnS catalyst for electroreduction process. Some CO_2_ will concurrently react with the strongly alkaline 1.0 m KOH to form carbonate or bicarbonate.^[^ ^23^, ^25^, ^34^ ^]^ The carbonate species formation process consumed KOH and generated water as a by‐product, which reduces OH^−^concentration resulting in the pH change. This is in good agreement with the variations in pH after 1 h experiments in relation to the catholyte volumes (Figure 4b). The catholyte of 10 mL has the largest pH decreased from 13.55 to 13.30, which is corresponding to the drop in OH^−^ concentration from 0.355 to 0.199 m, consequently resulting in the significant drop in current density as shown in Figure 4a. We performed a control experiment by continuously purging CO_2_ into the 1 m KOH without applied electrochemical potential (Figure S17d, Supporting Information). The observed drop in pH value over the time further confirmed the reaction of CO_2_ with KOH to form carbonate or bicarbonate that influences the electrolyte property. This justified the employment of 1.5 L of 1.0 m KOH in the long term stability test of SnS/GDL, ensuring the buffered OH^−^ concentration (Figure 2h).

### 2.4 The Role of Sulfur in the CO_2_RR of SnS

**Figure 5:**
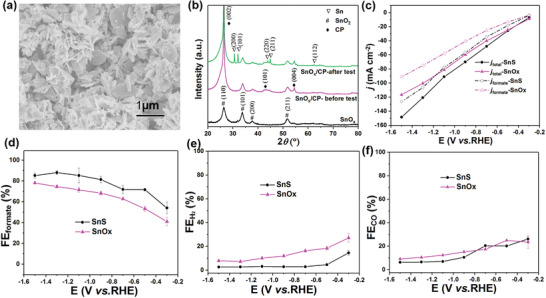
Effects of sulfur element in SnS catalyst. a) A SEM image of SnO*x*/GDL. b) XRD patterns of SnO*x*, SnO*x*/GDL‐before test, and SnO*x*/GDL‐after test. c) Steady‐state current density of SnO*x*/GDL and SnS/GDL samples, solid symbols represent the total current density (*j*_total_), while empty symbols represent the partial current density of formate (*j*_formate_). d–f) Faradic efficiencies toward formate, H_2_ and CO on SnO*x*/GDL and SnS/GDL samples. All the performance tests were conducted in the electrolyte of 1 m KOH. The error bars represent the standard deviations of three independent measurement of the sample.

It was reported that the incorporation of sulfur on Sn either as major, or adventitious additive as a doping element could enhance the CO_2_ electroreduction performance as it may help stabilize the intermediate *OCHO intermediate,^[^ ^14^ ^]^ weaken the interaction between CO and electrode,^[^ ^21a^ ^]^ and/or increase the catalysis sites by introducing atom distortion.^[^ ^19^ ^]^ To understand the role of sulfur in this alkaline system toward CO_2_RR performance, the sulfur element in the synthesized SnS was removed by thermal treatment in an oven at 500 °C for 2 h in air. As shown in SEM image in **Figure** **5**a and Figure S18 (Supporting Information), the obtained tin oxide (SnO*x*) retained the nanosheet morphology. XRD spectra in Figure 5b and Figure S18a (Supporting Information) displays characteristic peaks of SnO_2_ at (110), (101), (200), and (211), consistent with tetragonal rutile phase of SnO_2_ (JCPDS 21‐1250). Raman modes of SnOx in Figure S18b (Supporting Information) further indicate the oxide phase of SnO_2_. The SnO*x* was subsequently air‐brushed on a GDL with the loading of 1 mg cm^−2^. In comparison to SnS catalyst, the SnO*x* exhibits lower steady state current densities (Figure 5c), as well as ≈10% smaller FE_formate_ than that of SnS (Figure 5d). As shown in Figure 5f in comparison to SnO*x*, the lower FE_H2_ in SnS suggests sulfur acts to suppress hydrogen evolution. To identify the influence of alkaline electrolyte on SnOx, the performance of SnOx in 1 m KOH and 0.5 m KHCO_3_ electrolytes were compared in Figure S20 (Supporting Information). Both the selectivity and the current density can be greatly improved by alkaline electrolyte. However, SnS catalyst shows greater performance enhancement in comparison to SnO*_X_* (see Figure 2).

The Tafel plots of SnS and SnO*x* in KOH (Figure S14, Supporting Information) are 124 and 138 mV dev^−1^, respectively. The slightly lower Tafel slope of SnS illustrates that sulfur may promote the formation of absorbed *CO_2_ (RDS) by offering faster kinetics, which facilitates the formate production. The suppression of hydrogen evolution can be explained by the mechanism reported by Wang et al.^[^ ^35^ ^]^ Sulfur elements can suppress hydrogen evolution by activation of water and the formed hydrogen species preferred to react with adsorbed CO_2_ to form formate intermediate instead of following the hydrogen evolution pathway. Comparatively, Sn peaks at (200), (101), (220), (211), and (112) were seen after 1 h CO_2_ electroreduction for SnO_2_ (Figure 5b), indicating oxide phase is less stable and readily reduced to metallic Sn in tetragonal phase (JCPDS 04‐0673).

### 2.5 Proposed Mechanism of Alkaline Enhancement

**Figure 6:**
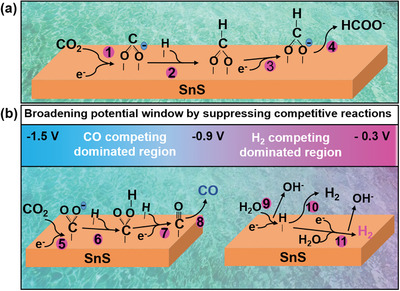
The diagrams proposed mechanistic aspects of electrochemical CO_2_ reduction to a) formate, and b) H_2_ and CO on SnS/GDL in alkaline KOH electrolyte.

There are three competing reactions in SnS‐based electrochemical CO_2_ reduction, namely formate, H_2_ and CO evolutions. The plausible pathways are summarized in **Figure** **6**. Our discussion emphasizes on how alkalinity influences the dynamic of those competing reactions, which contributes positively in broadening the potential window for formate production as we observed from our experimental data. As schematically illustrated in Figure 6a, the RDS for the formate generation is the transfer of an electron to the surface adsorbed *CO_2_ radical anion (step 1), follow by the protonation (step 2).^[^ ^3^, ^31^ ^]^ After the reduction of the *OCHO intermediate (step 3), the formate can readily release as a product (step 4). In this study, the concentration of protons is low (≈10^−13.55^ m) in 1 m KOH electrolyte and thus the protons needed in each pathway is expected to be derived from the Volmer reaction (step 9): * + H_2_O + e^−^ → *H_ads_ + OH^−^, where * is the adsorption site.^[^ ^36^ ^]^

There are two dominant competitive products, H_2_ and CO, which could influence the formate conversion efficiency. From the obtained CO_2_ electroreduction performance data presented in Figure 2f,g, the competing reactions can be divided into two regions: 1) H_2_ evolution competing dominated region occurred at a less negative potential range (≈−0.3 to −0.9 V vs RHE), and 2) CO evolution competing dominated region at a more negative potential range (≈−0.9 to −1.5 V vs RHE). In the H_2_ competing dominated region, after the Volmer reaction, the hydrogen was generated either following the Tafel (step 10):2 *H_ads_ → H_2_ + 2 * or Heyrovsky (step 11) :*H_ads_ + H_2_O + e^−^ → * + H_2_ + OH^−^ steps.^[^ ^36a^ ^]^ It is worth noting that although less significant H_2_ competition at −0.9 to −1.5 V, it still accounts for ≈10–25% suppression of hydrogen evolution reaction (see Figure 2f) in 1 m KOH in comparison to 0.5 m KHCO_3_ which understates the critical impact of hydrogen suppression across whole potential range. In an alkaline electrolyte, multiple reaction steps, and the slower kinetics of water dissociation result in about two orders of magnitude lower hydrogen evolution activity than that in acidic or neutral medium.^[^ ^36^, ^37^ ^]^ Therefore, as is consistent with our experimental data in Figure 2e,f, increasing electrolyte alkalinity (e.g., KOH of 0.1–1.0 m) successfully suppresses hydrogen evolution, which favors formate production.

In the CO competing dominated region, it is well documented that carbon atom in adsorbed *CO_2_^−^ radical anion is bonded to the catalyst surface (step 5) and protonation process occurs at the oxygen atom (step 6). The CO can be generated by further protonation on the *COOH intermediate (steps 7 and 8). The selectivity of CO_2_RR is determined by the active energy barrier for the formation of *OCHO (intermediate to formate) and *COOH (intermediate to CO).^[^ ^3^, ^38^ ^]^ According to Gabardo et al.,^[^ ^39^ ^]^ highly concentrated KOH can destabilize hydronium ions, which makes the reaction energy barrier for the *COOH intermediate much higher than that of *OCHO. Thus, increasing alkalinity makes formate production process more favorable. The suppression of CO and H_2_ at both competing regions broadens the electrochemical potential window for formate production from CO_2_.

## 3 Conclusions

We have demonstrated the unexpected significant broadening of an electrochemical potential window for CO_2_ reduction to formate on a SnS catalyst at high current densities using an alkaline electrolyte in a flow‐cell configuration. A wide potential window of −0.5 to −1.5 V vs RHE with FE_formate_ over 70% was achieved in 1 m KOH. The maximum FE_formate_ of 88 ± 2% was obtained at −1.3 V vs RHE and the current density can reach ≈148 mA cm^−2^ at −1.5 V. Based on the experimental data, it is evidenced that the suppression of H_2_ competing reaction in KOH medium occurred across the whole potential range, though more predominant at the less negative potentials. This is in addition to CO suppression at a more negative potential resulting in a widening of overall potential windows for formate production. A control experiment study indicated sulfur in SnS acts to suppress H_2_ generation as in comparison to SnO*x*. To ensure the long‐term current stability of the electrolysis reaction in KOH solution, we demonstrated the importance of buffering hydroxide concentration, which otherwise diminishes due to chemical reactions between KOH and CO_2_ to form carbonate or bicarbonate species. This study offers insights into the essence of integrating a suitable catalyst, cell design, and electrolyte toward achieved desirable CO_2_ electroreduction performance with features such as high formate conversion efficiencies at high current densities over a wide potential window. The outstanding performance obtained in this work may be extendable to other catalysts for formate conversion, which brings it closer to translational research of industrial relevant high yield formate production from CO_2_.

## 4 Experimental Section

### Chemicals and Materials

Tin (II) chloride (99%, Sigma‐Aldrich), ethylene glycol (Chem‐Supply), thiourea (99%, Sigma‐Aldrich), gas diffusion layer (Sigracet 39 BC, FuelCellStore), Carbon dioxide (CO_2_, 99.99%), hydrogen (H_2_, 99.99%), and Ar cylinders were purchased from BOC.

### Preparation—Catalyst Preparation

1 mmol of SnCl_2_ and 2 mmol of thiourea were dissolved in 35 mL of ethylene glycol and stirred for 1 h to obtain a homogeneous solution. The solution was then transferred to autoclave (50 mL). The autoclave was kept at 140, 160, 180, and 200 °C for 12 h. After cooling down to room temperature, the precipitates were collected and washed by centrifuging with ethanol for 5 times and dried to get the tin sulfide particles. The particles were exfoliated to nanosheets by ultrasonicating in isopropanol to obtain a homogenous SnS catalyst suspension (25 mg mL^−1^).

### Preparation—Working Electrode Preparation

400 µL of SnS catalyst suspension, 460 µL of isopropanol, 100 µL of DI water, and 40 µL of nafion solution (15%) were ultrasonically mixed for 30 min to form a homogenous catalyst ink. The different volumes of catalyst ink were air‐brushed on the gas diffusion layers (GDL) and dried at 50 °C overnight to serve as SnS/GDL working electrodes.

### Characterization

XRD patterns of the tin sulfide based samples were collected on a PANalytical Empyrean diffractometer with Cu K_*α*_ radiation at a scan rate of 2° min^−1^. The surface morphologies of the samples were recorded on a SEM of JEOL JSM‐7500FA. TEM was carried out on a JEOL JEM‐2100F microscope. The XPS measurements were performed on the VG Multilab 2000 (VG Inc.) photoelectron spectrometer with the monochromatic Al K_*α*_ radiation under vacuum at 2 × 10^−6^ Pa. Raman analysis was performed with a Raman spectrometer of HORIBA Scientific with the laser line of 633 nm and the accumulations of 50.

### Electrochemical Characterization

All the measurements were carried out on a CHI660D potentiostat at room temperature, in a home‐made flow‐cell separated by a cation exchange membrane (Nafion 115) in the neutral electrolyte and anion exchange membrane (Fumasep FAB‐PK‐130, FuelCellStore) in the alkaline electrolyte. HgO electrode and nickel mesh were used as the reference and counter electrodes, respectively, when employing alkaline electrolyte. Ag/AgCl (3 m NaCl) electrode and Pt mesh were used as the reference and counter electrodes, respectively, when employing neutral electrolyte (0.5 m KHCO_3_). The CO_2_ gas was introduced into the cathodic chamber at a flow rate of 20 mL min^−1^ and the electrolyte was pumped by a peristaltic pump (BT100‐2J, Thermoline) at the flow rate of 17.5 mL min^−1^. All tests were conducted without IR‐compensation.

### Product Analysis

The gaseous products were analyzed by a gas chromatography (GC) (8610C, SRI Instruments) equipped with both flame ionization detector (FID) and thermal conductivity detector (TCD). CO, CH_4_, C_2_H_4_, C_2_H_6_ were detected by the FID and H_2_, CO, CH_4_, C_2_H_4_, C_2_H_6_, and CO_2_ were detected by TCD. The analysis of liquid products was carried out on a 400 MHz NMR spectrometer (Bruker Avance). The 1D ^1^H spectra were measured with water suppression. 1‐propanesulfonic acid 3‐(trimethylsilyl) sodium (DSS) was used as internal standard solution. A 0.5 mL of product‐containing electrolyte, 0.1 mL of DSS (99.7%, Sigma‐Aldrich), and 0.1 mL of D_2_O (99.9%, Cambridge Isotope Lab) was added in the NMR tube and mixed by ultrasonication before NMR analysis.

### Electrochemically Active Surface Area (ECSA) Measurement

The ECSA of SnS was obtained using cyclic voltammetry over the potential window of −0.5 to −0.7 V in the electrolyte of 1 m KOH. The *C*_dl_ was estimated by plotting the ∆*j* (*j*_a_–*j*_c_) at 0.6 V against the scan rate. The specific capacitance (20–60 µF cm^−2^) of 40 µF cm^−2^ was used to calculate the ECSA^[^ ^40^ ^]^

(1) $$\mathrm{ECSA}=C_{\mathrm{dl}}/40\;\mu F\cdot cm^{-2}$$

## Conflict of Interest

The authors declare no conflict of interest.

## Supporting information

Supporting Information

## Data Availability

Data available on request from the authors.
