# Supplementary material for: Boosting Formate Production from CO2 at High Current Densities Over a Wide Electrochemical Potential Window on a SnS Catalyst
Source: Adv Sci (Weinh). 2021 May 29;8(15):2004521. doi: 10.1002/advs.202004521 (PMC8336617; doi:10.1002/advs.202004521)
Supplement: Supplementary file 1 — Supporting Information [file ADVS-8-2004521-s001.pdf]

WILEY-VCH

© 2021 Wiley-VCH GmbH

ADVANCED SCIENCE
Open Access

Supporting Information

for *Adv. Sci.*, DOI: 10.1002/advs.202004521

Boosting Formate Production from CO_2 at High Current Densities over a wide Electrochemical Potential Window on a SnS Catalyst

Jinshuo Zou, Chong-Yong Lee and Gordon G. Wallace**

Supporting Information

Boosting formate production from CO_2 at high current densities over a wide electrochemical potential window on a SnS catalyst

Jinshuo Zou, Chong-Yong Lee and Gordon G. Wallace**

ARC Centre of Excellence for Electromaterials Science,
Intelligent Polymer Research Institute,
AIIM, Innovation Campus,
University of Wollongong,
Wollongong, NSW 2500,
Australia.

E-mail: cylee@uow.edu.au; gwallace@uow.edu.au

Table S1. Performance comparison of different Sn-based electrocatalysts for CO_2 reduction to formate from recent literatures.

Electrocatalyst	Electrolyte	Setup	Products	Formate FEmax	j at the potential of FE_{max}	Ref
Sn quantum sheets	0.1 M $NaHCO_3$	H-cell	$HCOO^-$	89% (-1.134V vs. RHE)	21.1 mA cm^{-2}	[1]
Sn-CF1000	0.1 M $KHCO_3$	H-cell	$HCOO^-$ & CO	65% (-0.8V vs. RHE)	11 $mAcm^{-2}$	[2]
SnOx	0.5 M $KHCO_3$	H-cell	$HCOO^-$	87.1% (-1.6V vs SHE)	14.0 $mAcm^{-2}$	[3]
SnO_2 Wire in Tube	0.1 M $KHCO_3$	H-cell	$HCOO^-$ & CO	~70%(-1.29V vs RHE)	12 mA cm^{-2}	[4]
SnO_2 Porous Nanowires	0.1 M KH CO_3	H-cell	$HCOO^-$ & CO	78% (−1.0 V vs. RHE)	10 mA cm^{-2}	[5]
Ultra-small SnO_2	1 M KOH	Flow-cell	$HCOO^-$, C_2H_4, C_2H_5OH, CH_3COOH, n-C_3H_7OH	74% (−0.73 V vs. RHE)	~75 mA cm^{-2}	[6]
Ultra Small SnO Nanoparticles	0.5 M $KHCO_3$	H-cell	$HCOO^-$ & CO	68% (−0.73 V vs. RHE)	20 mA cm^{-2}	[7]
Sn-GDE	0.5 M $NaHCO_3$	Flow-cell	$HCOO^-$ & CO	71% (−1.1V vs. RHE)	8.58 mA cm^{-2}	[8]
SnS_2/rGO	0.5 M $NaHCO_3$	H-cell	$HCOO^-$ & CO	84.5 (-0.788V vs. RHE)	~13.9 mA c m^{-2}	[9]
Nano SnO_2	0.1 M $NaHCO_3$	H-cell	$HCOO^-$ & CO	86.2% (−1.134V vs. RHE)	5.4 mA cm^{-2}	[10]
SnO_2 nanosheets on carbon cloth	0.5 M $KHCO_3$	H-cell	$HCOO^-$ & CO	87±2 % (-0.988V vs. RHE)	~50 mA cm^{-2}	[11]
SnS_2 monolayer	0.1 M $KHCO_3$	H-cell	$HCOO^-$ & CO	94 ± 5% (-0.8V vs. RHE)	~46 mA cm^{-2}	[12]
Electrodeposited Sn	0.1 M $KHCO_3$	H-cell	0.1 M KHCO3	91.7% (−0.734V vs. RHE)	0.9–1.4 mA cm^{-2}	[13]
SnS/GDL	1 M KOH	Flow-cell	$HCOO^-$ & CO	88.10±1.87% (-1.3V vs. RHE)	120.63 mA cm^{-2}	This work

Note: The potentials were converted to RHE scale based on the equations: E (vs. RHE) = E (vs. Ag/AgCl) + 0.0591 * pH + 0.21 and E (vs. RHE) = E (vs. SCE) + 0.0591 * pH + 0.24. It was assumed that the pH values of CO_2-saturated 0.1 M or 0.5 M $NaHCO_3$ or $KHCO_3$ aqueous solution is 6.8 and 7.2, respectively.

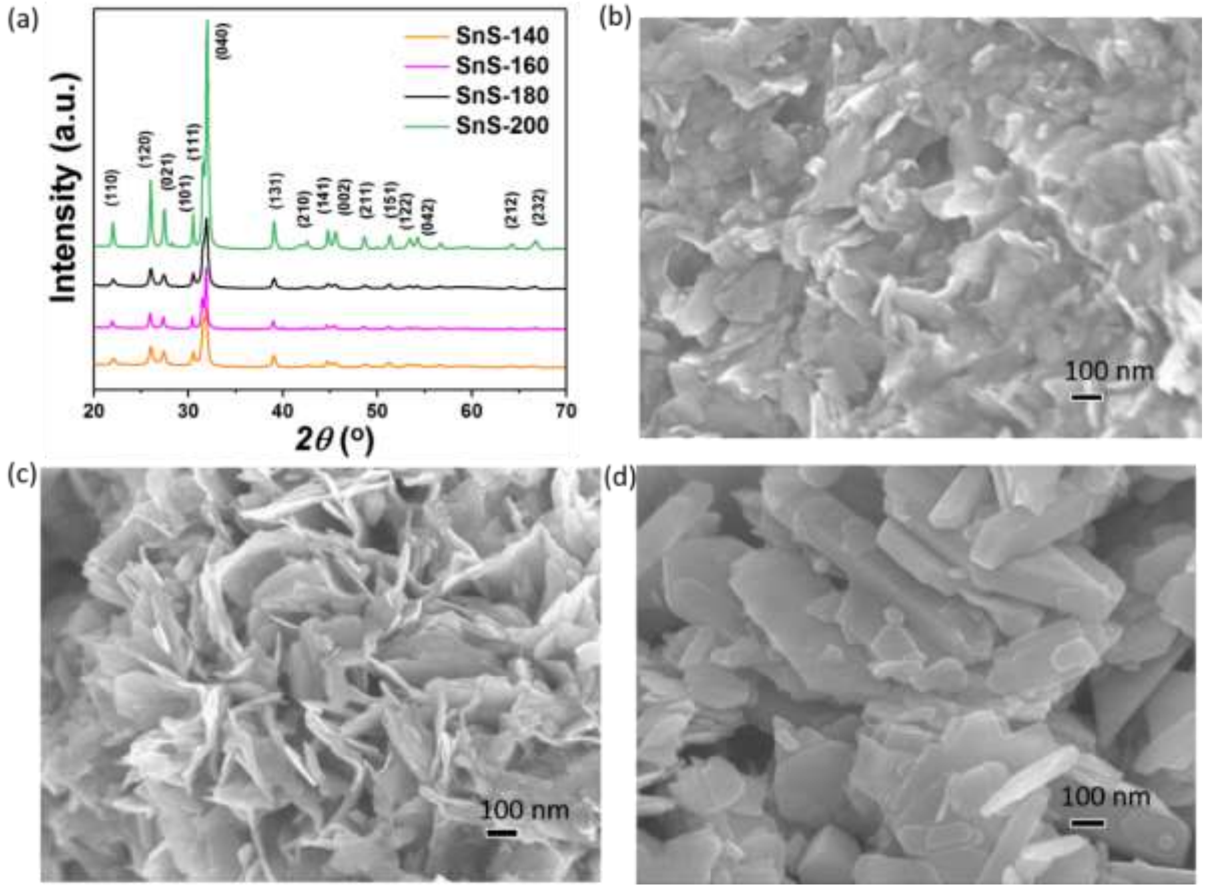

Figure S1. (a) XRD patterns of SnS catalysts obtained at different solvothermal synthesis temperatures from 140 to 200 ºC. SEM images of SnS heated at (b) 140 ^{o}C (SnS-140), (c) 160 ^{o}C (SnS-160), and (d) 200 ^{o}C (SnS-200).

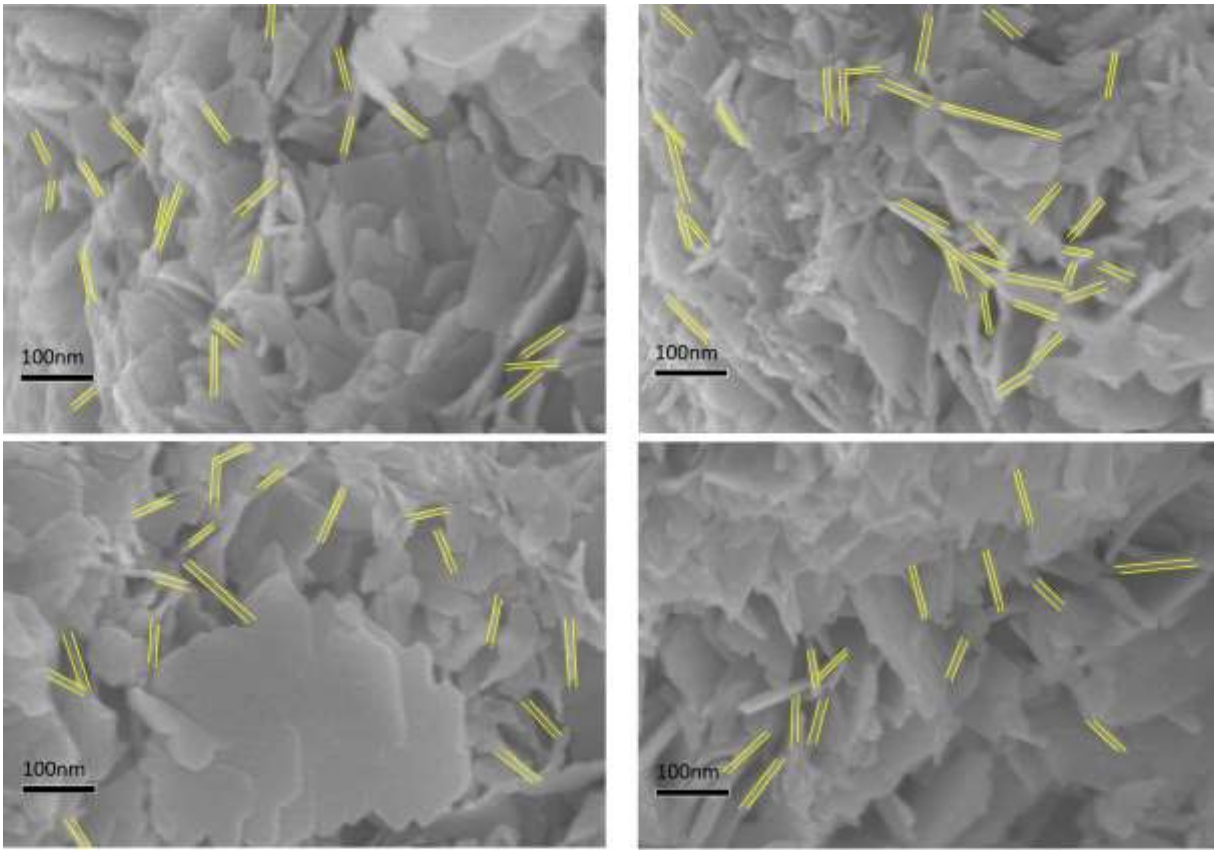

Figure S2. SEM images of SnS nanosheets. The parallel yellow lines marked the layer thicknesses.

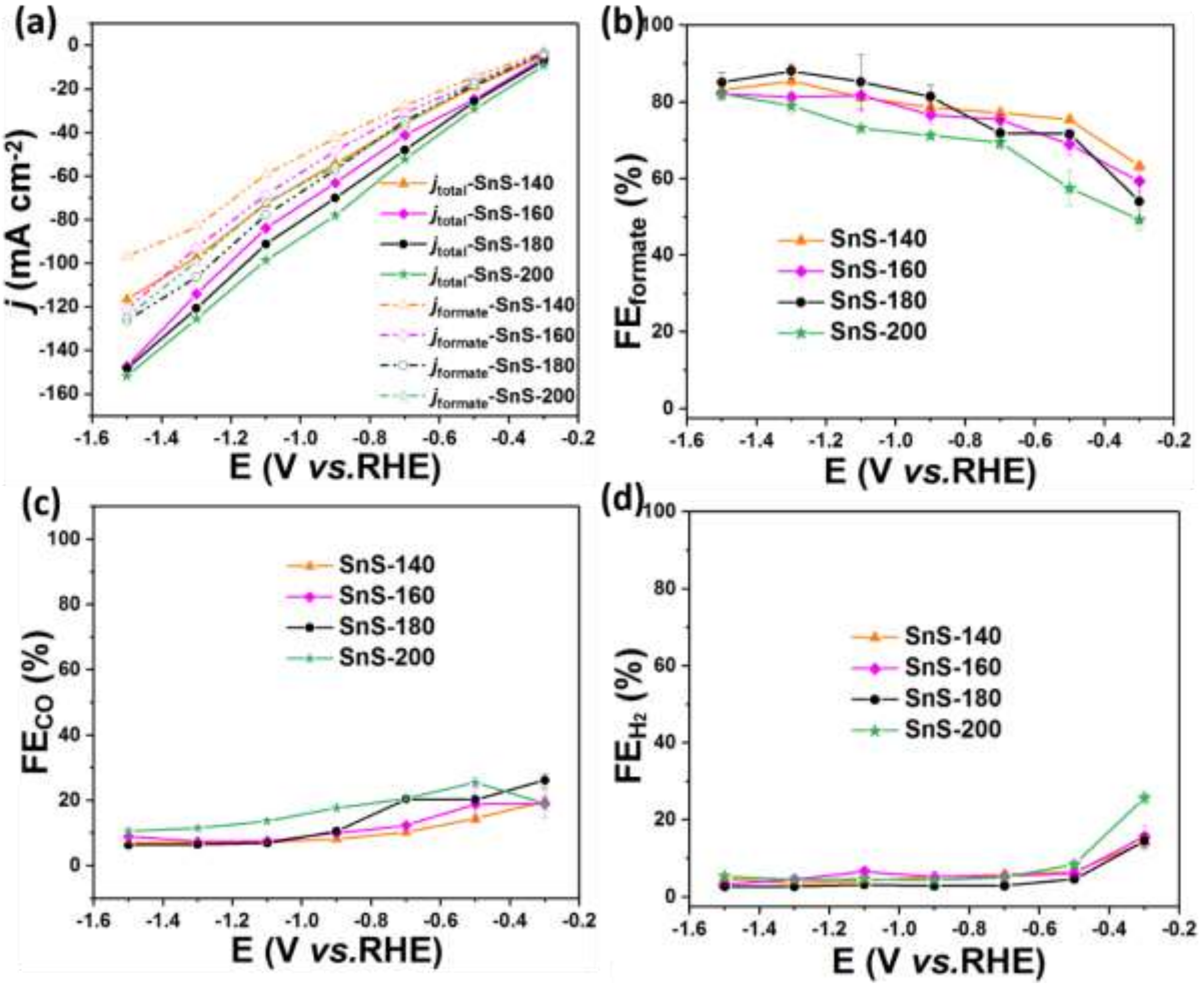

Figure S3. Steady-state current density (a), Faradic efficiencies towards formate (b), CO (c) and H_2 (d) productions from CO_2 electroreduction in 1 M KOH on SnS synthesis at differrent temperatures. The error bars represent the standard deviations of three independent measurements of the samples.

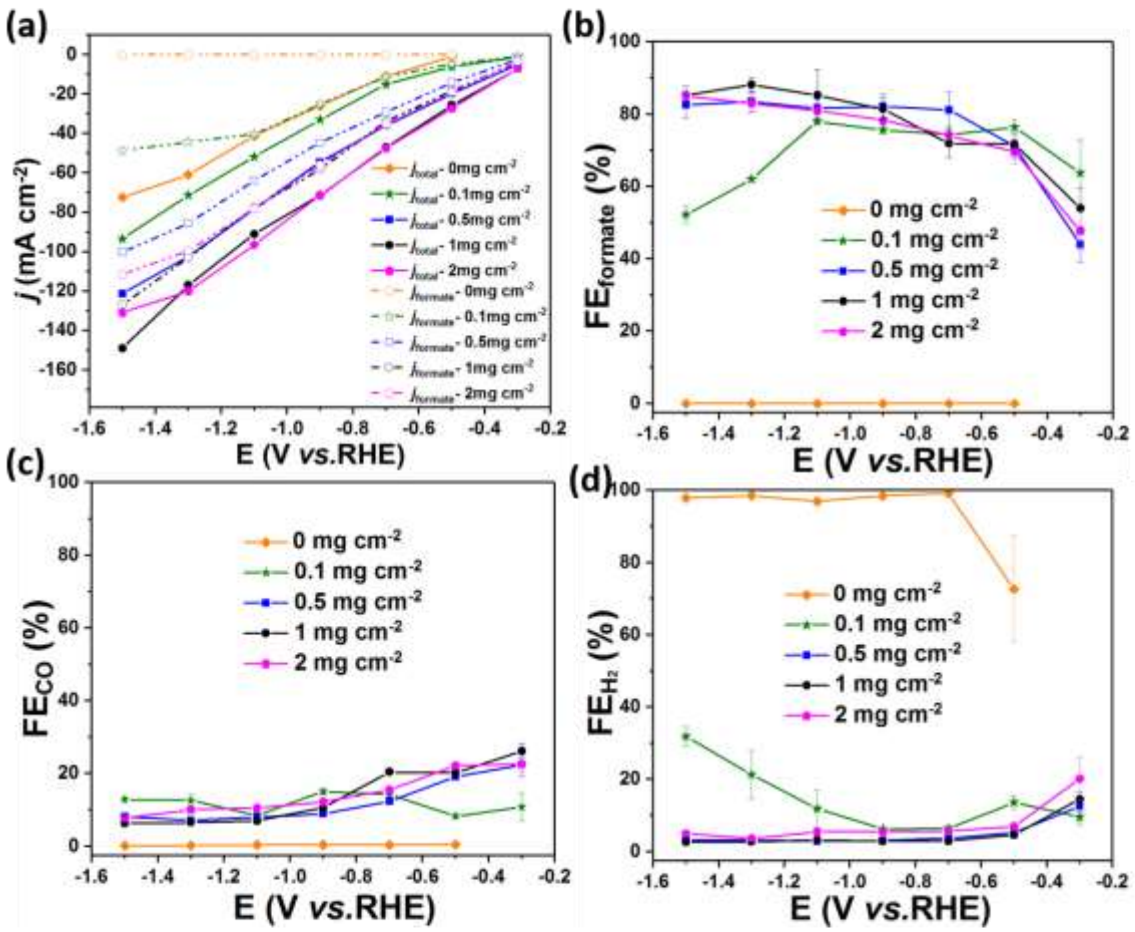

Figure S4. Steady-state current density (a), Faradic efficiencies towards formate (b), CO (c) and H_2 (d) productions from CO_2 electroreduction in 1 M KOH on SnS synthesized at 180 ^oC with different catalyst loading. The error bars represent the standard deviations of three independent measurement of the sample.

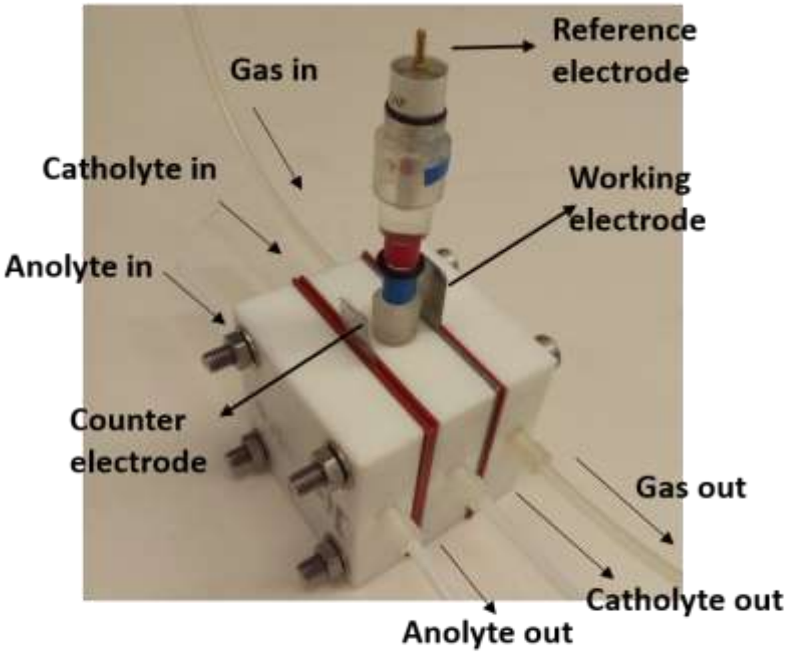

Figure S5. The photograph of a flow-cell for CO_2 eelctroreduction employed in this study.

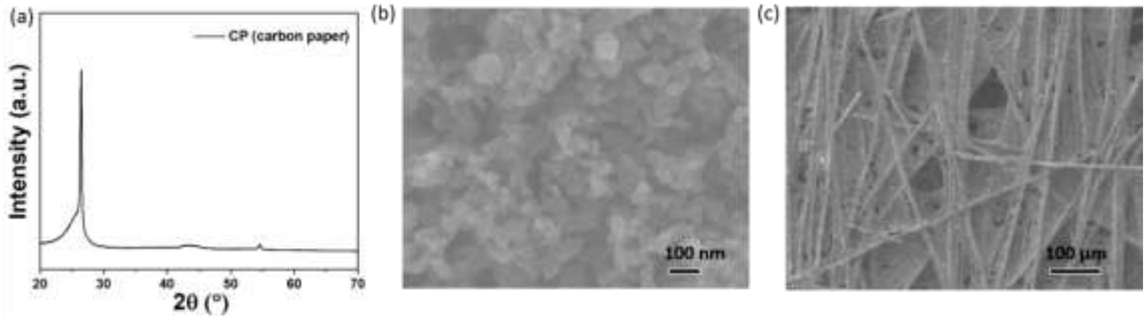

Figure S6. (a) XRD pattern and (b, c) SEM images of gas diffusion layer (GDL). The SEM image in (b) is the carbon based mesoporous layer side and the SEM image in (c) is the carbon fibre layer side.

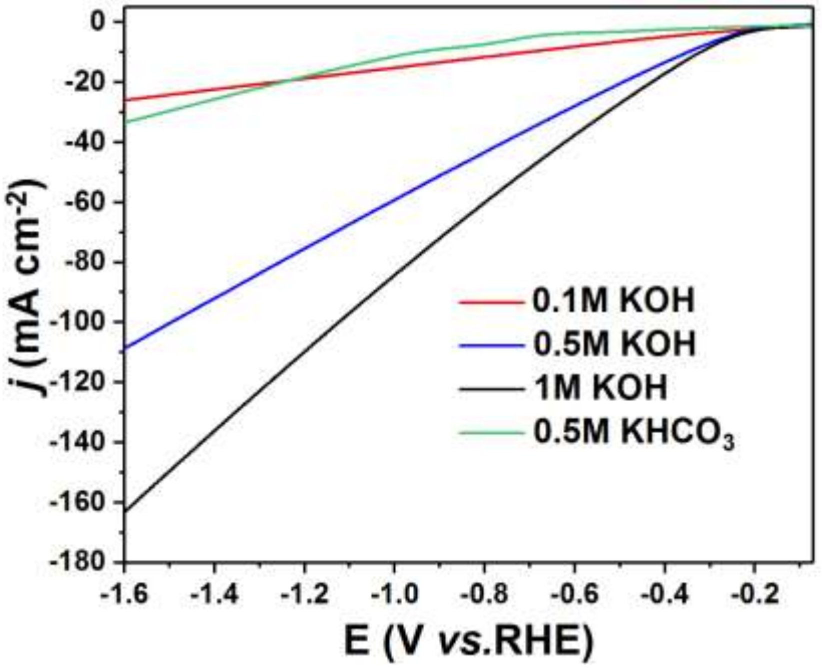

Figure S7. Linear Sweep Voltammetry (LSV) curves of SnS/GDL samples performed in different electrolytes at 10 mV s^{-1}.

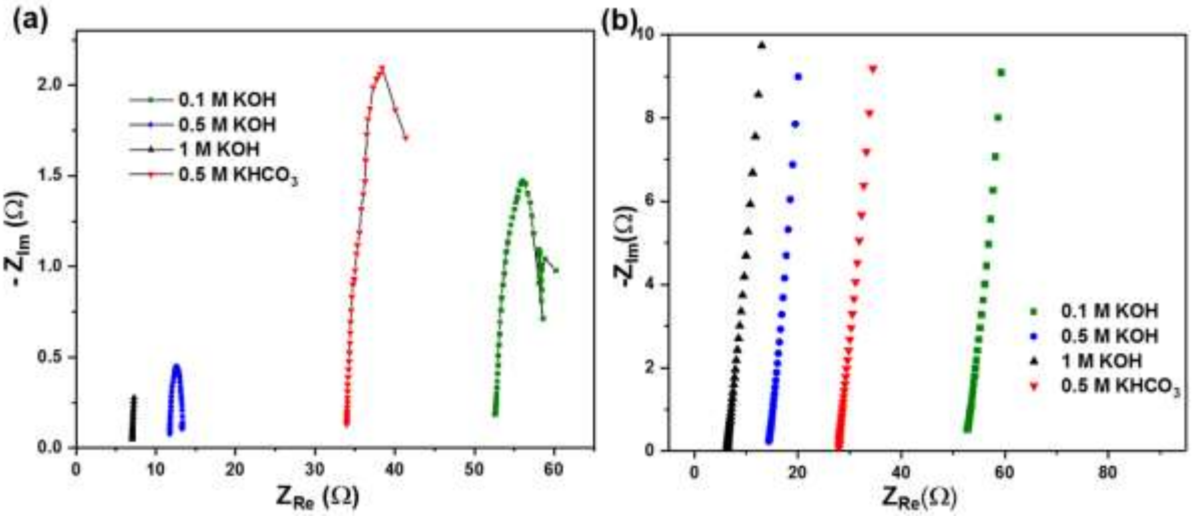

Figure S8. Nyquist impedance plots of SnS/GDL in different electrolytes obtained at -1.5V vs. RHE (a) and open circuit potentials (b).

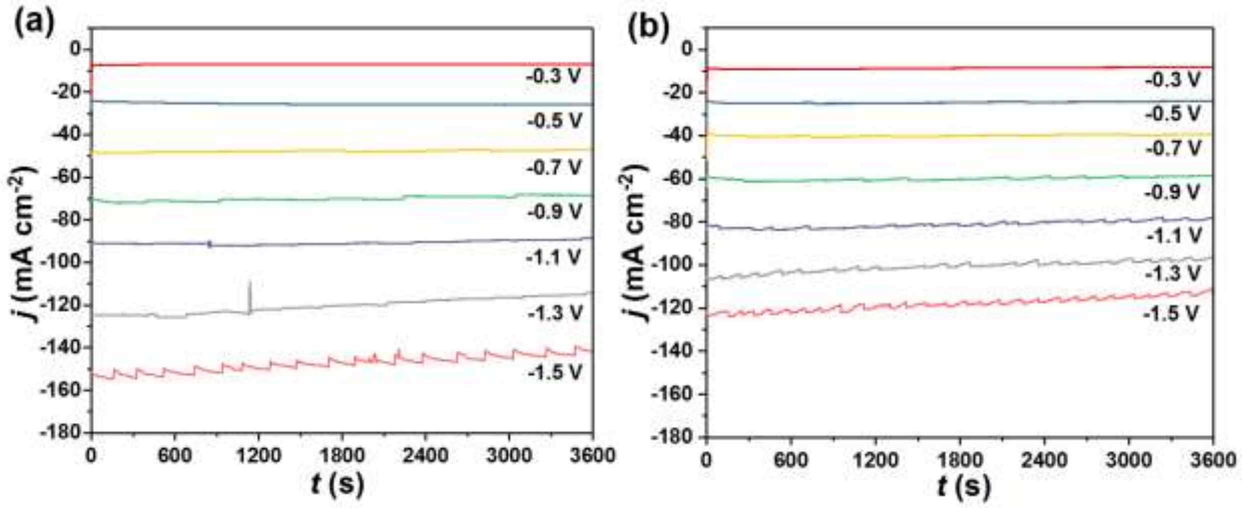

Figure S9. Amperometric i-t curves of (a) SnS and (b) SnOx at applied potentials from -0.3 to -1.5 V vs. RHE.

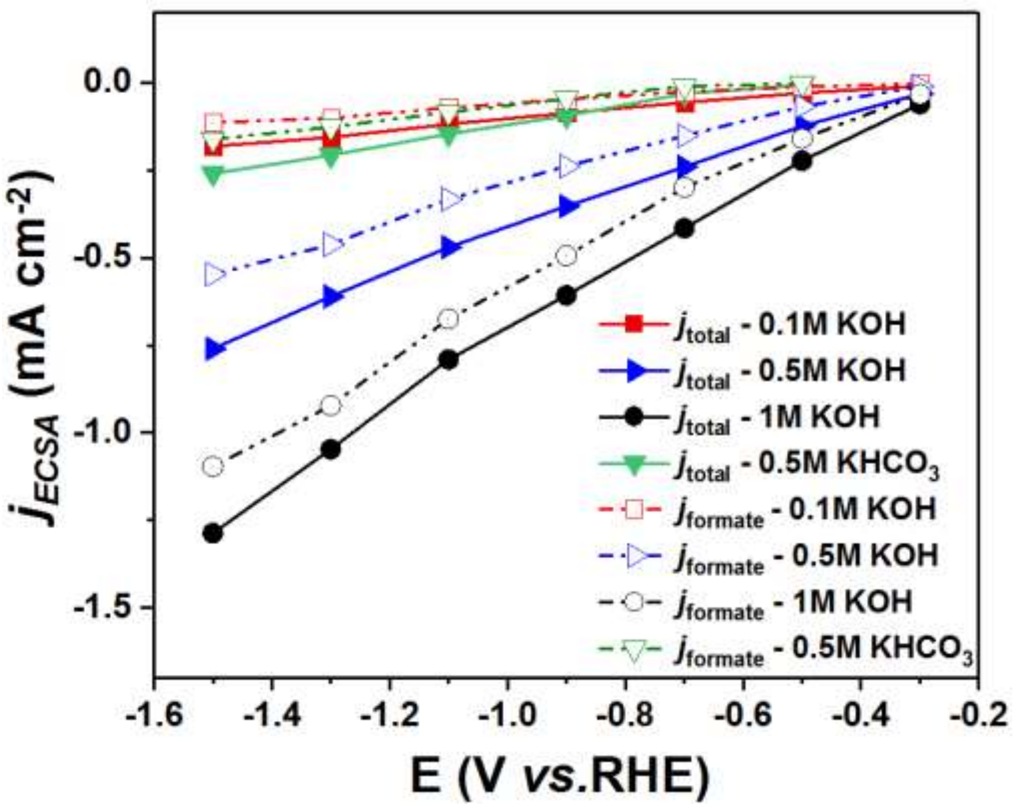

Figure S10. ECSA normalized steady-state current density of SnS/GDL sample at different potentials from -0.3 to -1.5 V vs. RHE.

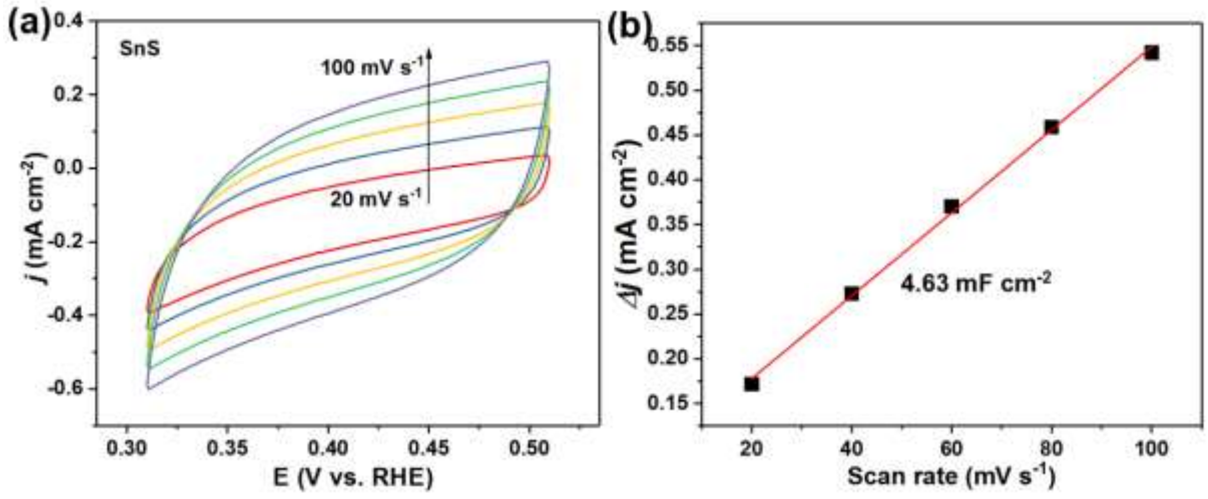

Figure S11. (a) CV curves of SnS, obtained at the non-Faradaic capacitance current range at the scan rates of 20, 40, 60, 80, and 100 mV s^{-1} in the electrolyte of 1 M KOH; (b) plot of the Δj - Scan rate derived from CV curves in (a).

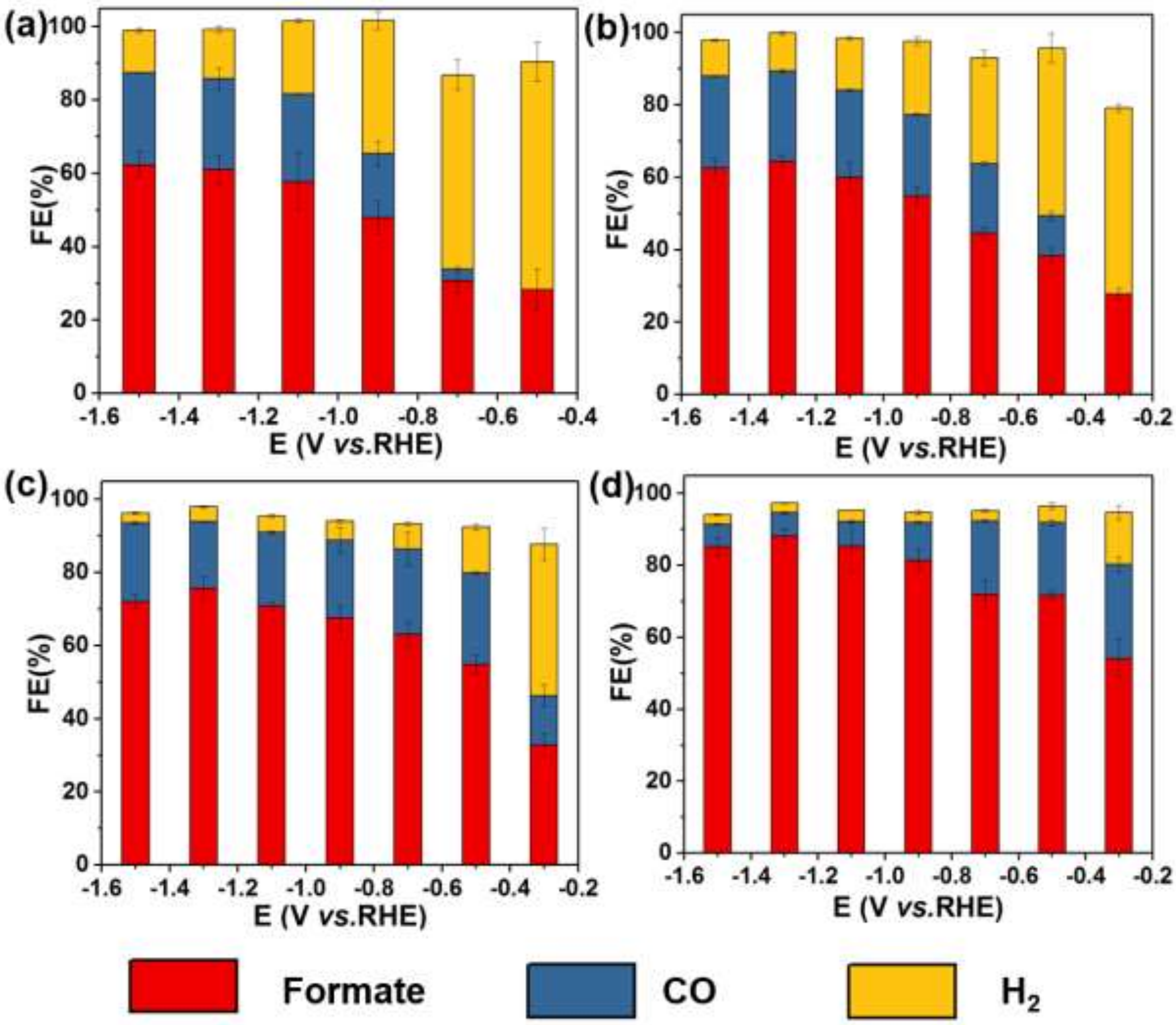

Figure S12. Total Faradic efficiency obtained in 0.5 M $KHCO_3$ (a), 0.1 M KOH (b), 0.5 M KOH (c), 1 M KOH (d). The error bars represent the standard deviations of three independent measurements of the samples.

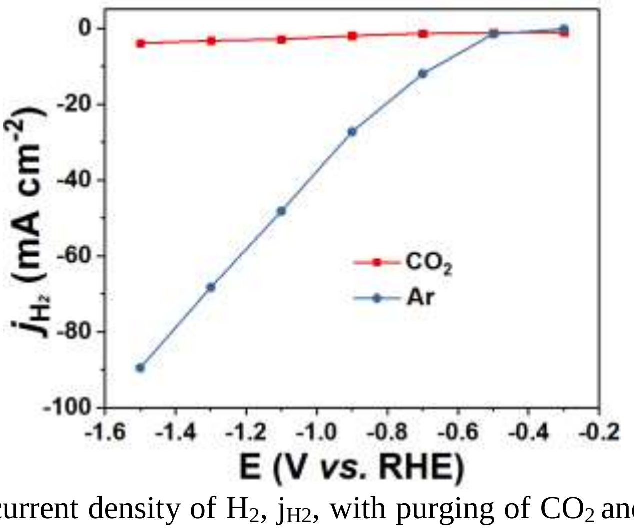

Figure S13. Partial current density of H_2, j_{H2}, with purging of CO_2 and Ar gas in 1 M KOH electrolyte.

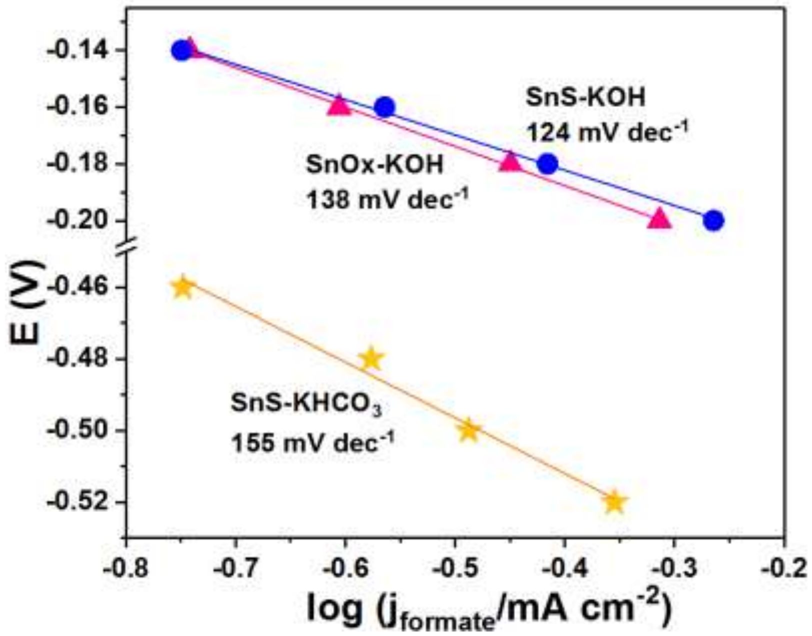

Figure S14. Tafel plots of SnS and SnOx catalysts in 1 M KOH electrolyte and SnS in 0.5 M $KHCO_3$ electrolyte

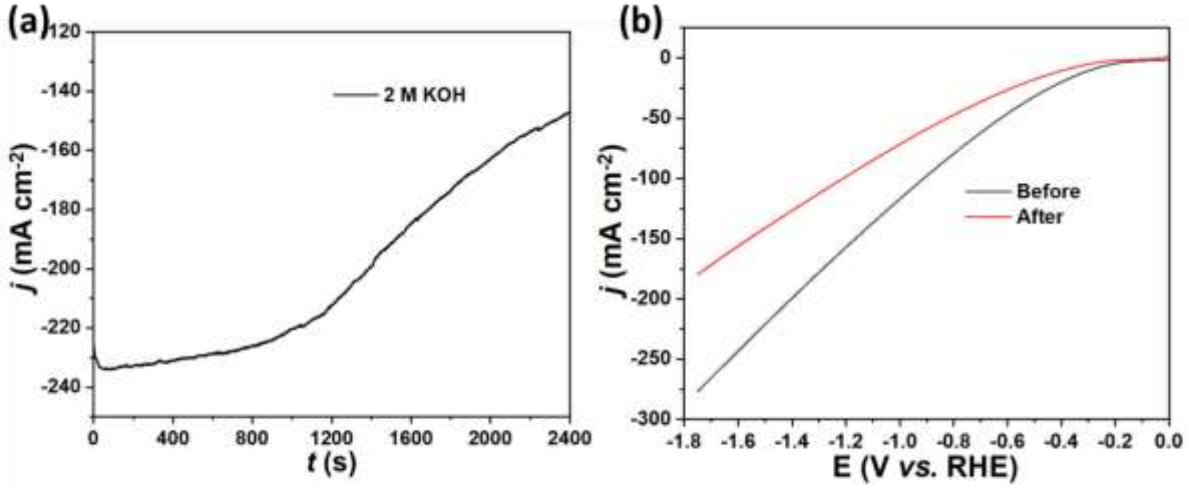

Figure S15. (a) j-t curve of CO_2 electroreduction on a SnS/GDL in 2 M KOH at -1.5 V vs. RHE, (b) LSV curves of SnS/GDL before and after 2400 s test at -1.5 V vs. RHE.

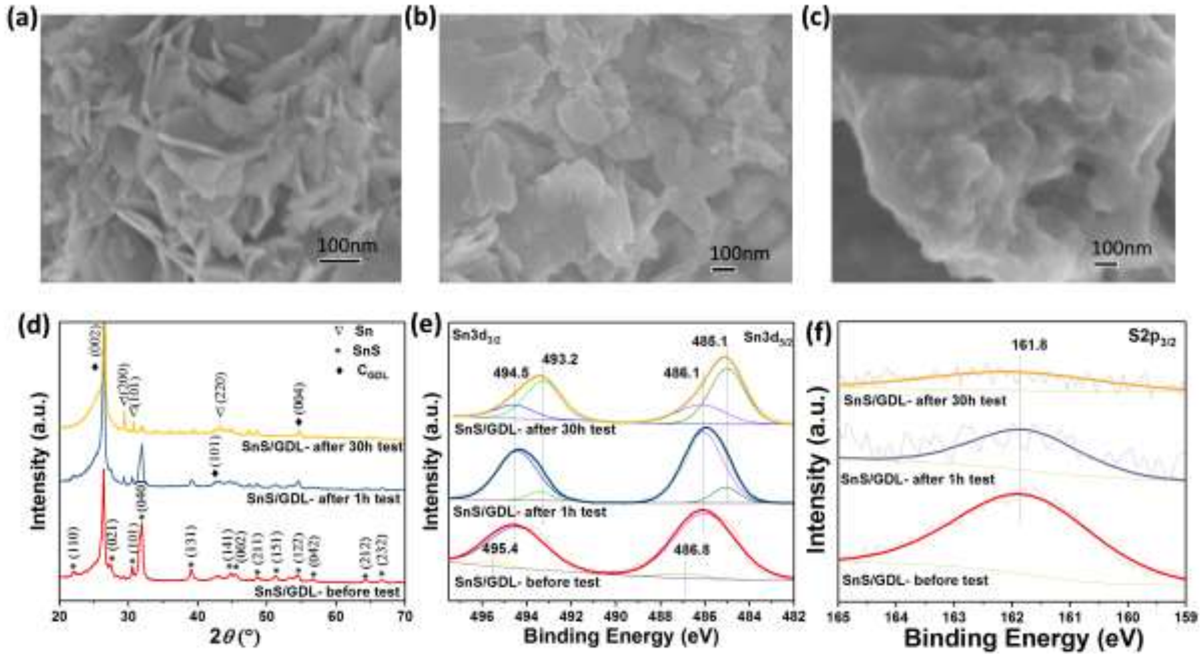

Figure S16. SEM images of (a) SnS/GDL before performance test, (b) SnS/GDL after 1 h performance test, (c) SnS/GDL after 30 h performance test, (d) XRD patterns of SnS/GDL before and after performance test, (e, f) XPS spectra of Sn and S elements of SnS/GDL before and after performance test.

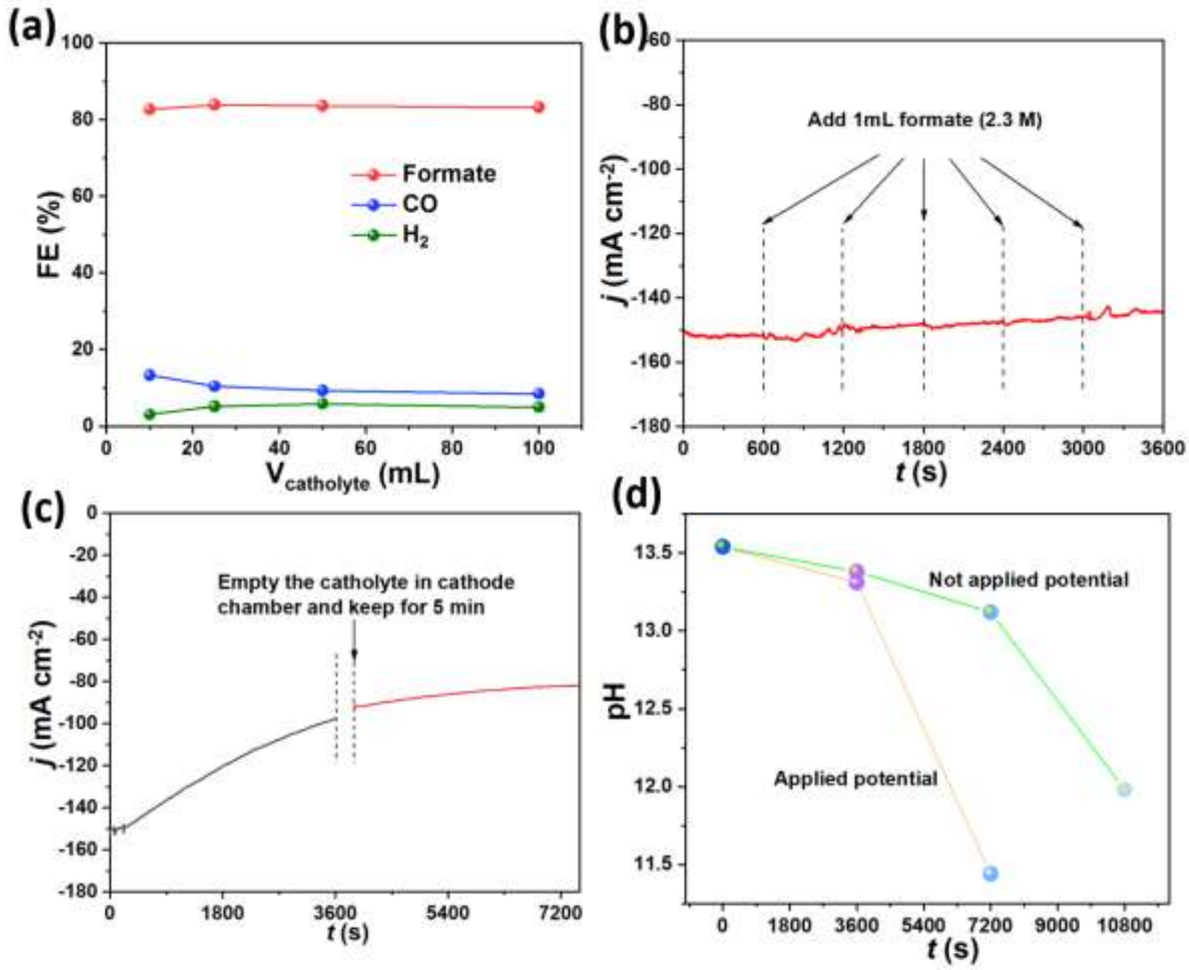

Figure S17. (a) Faradic efficiencies towards formate, CO, and H_2 productions from CO_2 electroreduction performed on SnS/GDL samples with different catholyte volumes. (b) Formate effect on the current density. The j-t curve with consecutively adding 1 mL of 2.3 M formate into the catholyte every 10 min. The experiment was conducted on a SnS/GDL working electrode in 50 mL of catholyte (1 M KOH) at the potential of -1.5 V vs. RHE. (c) CO effect on the current density. The experiment was conducted on a SnS/GDL working electrode in 10 mL of catholyte (1 M KOH) at -1.5 V vs. RHE. (d) The plot of pH change in catholyte chamber (10 mL KOH) as a function of time with continues CO_2 gas purging with (-1.5 V vs. RHE) or without applied potential.

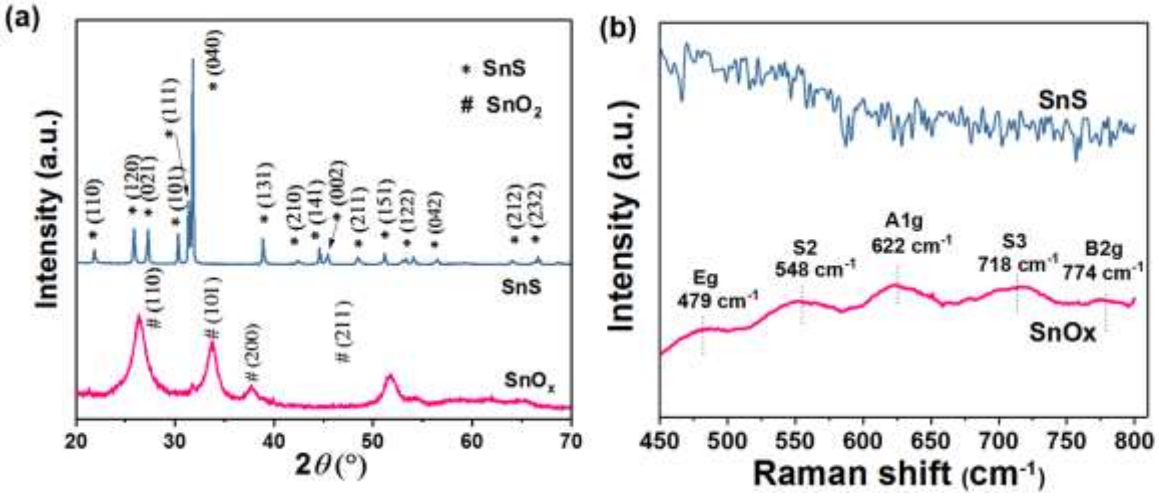

Figure S18. (a) XRD patterns and (b) Raman spectra of SnS and SnOx. The peaks at 497, 548, 622, 718, and 774 cm^{-1} are the Raman modes of SnO_2 [15].

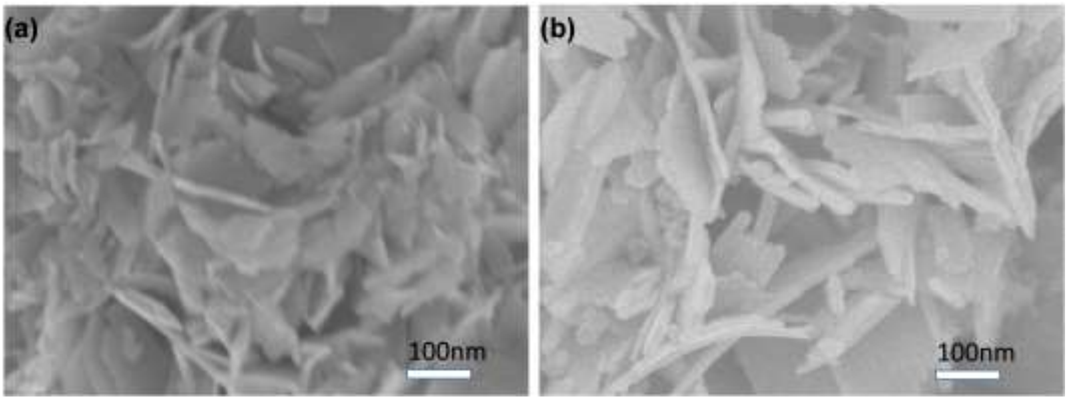

Figure S19. SEM images of (a) SnS and (b) SnOx samples.

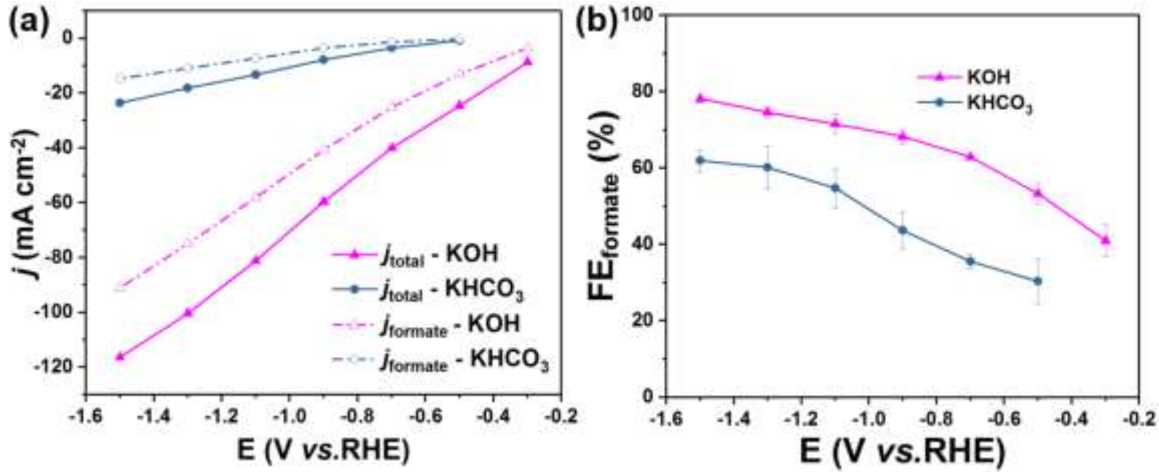

Figure S20. (a) Steady-state current density of SnOx/GDL in 1 M KOH and 0.5 M $KHCO_3$, solid symbols represent the total current density (j_{total}) while empty symbols represent the partial current density of formate ($j_{formate}$). (b) formate Faradic efficiency of SnOx/GDL in 1 M KOH and 0.5 M $KHCO_3$.

References

[1] F. Lei, W. Liu, Y. Sun, J. Xu, K. Liu, L. Liang, T. Yao, B. Pan, S. Wei, Y. Xie, Metallic tin quantum sheets confined in graphene toward high-efficiency carbon dioxide electroreduction, Nat. Commun., 7 (2016) 1-8.

[2] Y. Zhao, J. Liang, C. Wang, J. Ma, G.G. Wallace, Tunable and efficient tin modified nitrogen‐doped carbon nanofibers for electrochemical reduction of aqueous carbon dioxide, Adv. Energy Mater., 8 (2018) 1702524.

[3] Y. Li, J. Qiao, X. Zhang, T. Lei, A. Girma, Y. Liu, J. Zhang, Rational design and synthesis of SnOx electrocatalysts with coralline structure for highly improved aqueous CO_2 reduction to formate, ChemElectroChem, 3 (2016) 1618-1628.

[4] L. Fan, Z. Xia, M. Xu, Y. Lu, Z. Li, 1D SnO_2 with Wire‐in‐Tube Architectures for Highly Selective Electrochemical Reduction of CO_2 to C1 Products, Adv. Funct. Mater., 28 (2018) 1706289.

[5] B. Kumar, V. Atla, J.P. Brian, S. Kumari, T.Q. Nguyen, M. Sunkara, J.M. Spurgeon, Reduced SnO_2 porous nanowires with a high density of grain boundaries as catalysts for efficient electrochemical CO_2‐into‐HCOOH conversion, Angew. Chem. In. Ed., 56 (2017) 3645-3649.

[6] C. Liang, B. Kim, S. Yang, Y. Liu, C.F. Woellner, Z. Li, R. Vajtai, W. Yang, J. Wu, P.J. Kenis, High efficiency electrochemical reduction of CO_2 beyond the two-electron transfer pathway on grain boundary rich ultra-small SnO_2 nanoparticles, J. Mater. Chem. A, 6 (2018) 10313-10319.

[7] J. Gu, F. Héroguel, J. Luterbacher, X. Hu, Densely packed, ultra small SnO nanoparticles for enhanced activity and selectivity in electrochemical CO_2 reduction, Angew. Chem., 130 (2018) 2993-2997.

[8] E. Irtem, T. Andreu, A. Parra, M. Hernández-Alonso, S. García-Rodríguez, J. Riesco-García, G. Penelas-Pérez, J. Morante, Low-energy formate production from CO_2 electroreduction using electrodeposited tin on GDE, J. Mater. Chem. A, 4 (2016) 13582-13588.

[9] F. Li, L. Chen, M. Xue, T. Williams, Y. Zhang, D.R. MacFarlane, J. Zhang, Towards a better Sn: efficient electrocatalytic reduction of CO_2 to formate by Sn/SnS_2 derived from SnS_2 nanosheets, Nano Energy, 31 (2017) 270-277.

[10] S. Zhang, P. Kang, T.J. Meyer, Nanostructured tin catalysts for selective electrochemical reduction of carbon dioxide to formate, J. Am. Chem. Soc., 136 (2014) 1734-1737.

[11] F. Li, L. Chen, G.P. Knowles, D.R. MacFarlane, J. Zhang, Hierarchical mesoporous SnO_2 nanosheets on carbon cloth: a robust and flexible electrocatalyst for CO_2 reduction with high efficiency and selectivity, Angew. Chem. In. Ed., 56 (2017) 505-509.

[12] J. He, X. Liu, H. Liu, Z. Zhao, Y. Ding, J. Luo, Highly selective electrocatalytic reduction of CO_2 to formate over Tin (IV) sulfide monolayers, J. Catal., 364 (2018) 125-130.

[13] C. Zhao, J. Wang, Electrochemical reduction of CO_2 to formate in aqueous solution using electro-deposited Sn catalysts, Chem. Eng. J., 293 (2016) 161-170.

[14] Y. Chen, M.W. Kanan, Tin oxide dependence of the CO_2 reduction efficiency on tin electrodes and enhanced activity for tin/tin oxide thin-film catalysts, J. Am. Chem. Soc., 134 (2012) 1986-1989.

[15] P. Sangeetha, V. Sasirekha, V. Ramakrishnan, Micro‐Raman investigation of tin dioxide nanostructured material based on annealing effect, J. Raman Spectrosc., 42 (2011) 1634-1639.
